# Effects of pre-pregnancy dairy consumption on gestational diabetes mellitus: a prospective cohort study among Chinese women

**DOI:** 10.3389/fnut.2026.1769975

**Published:** 2026-04-29

**Authors:** Danting Li, Jing He, Wenjuan Xie, Jing Yang, Lili Wang, Dan Zhao, Nana Zhang, Ruonan Duan

**Affiliations:** 1 Department of Clinical Nutrition, Shanxi Bethune Hospital, Shanxi Academy of Medical Sciences, Tongji Shanxi Hospital, Third Hospital of Shanxi Medical University, Taiyuan, Shanxi, China; 2 Tongji Hospital, Tongji Medical College, Huazhong University of Science and Technology, Wuhan, Hubei, China; 3 Department of Obstetrics, Shanxi Bethune Hospital, Shanxi Academy of Medical Sciences, Tongji Shanxi Hospital, Third Hospital of Shanxi Medical University, Taiyuan, Shanxi, China; 4 Department of Clinical Nutrition, Taiyuan Central Hospital, Taiyuan, Shanxi, China

**Keywords:** dairy consumption, gestational diabetes mellitus, pre-pregnancy, prospective cohort, yogurt

## Abstract

**Background:**

The consumption of dairy products has been suggested to be associated with the prevention of obesity, cardiovascular diseases, and type 2 diabetes. However, it is unclear whether dairy consumption has a protective effect on gestational diabetes mellitus (GDM). This study aimed to investigate the prospective associations between pre-pregnancy dairy consumption and risk of GDM among Chinese women.

**Methods:**

A total of 1,012 women aged 18–40 years were enrolled from a prospective cohort between 2022 and 2024. Dairy consumption 1 year before pregnancy was collected by food frequency questionnaire. To screen for GDM, participants were scheduled for an oral glucose tolerance test (OGTT) at 24–28 weeks of gestation. Logistic regression and restricted cubic spline analyses were conducted to analyze the associations between dairy consumption and risk of GDM.

**Results:**

During the follow-up, 126 (12.5%) were diagnosed with GDM. Compared with non-consumers of room-temperature-storage yogurt, women in the highest tertile of consumption had 2.64-fold higher odds of GDM (OR: 2.64, 95% CI: 1.25–5.38) after adjusting for potential confounders. In subgroup analyses, the positive associations between room-temperature-storage yogurt consumption and risk of GDM were observed in women who were 30 years and older, whose pre-pregnancy BMI was lower than 24.0 kg/m^2^, and who were multiparas. No significant association was found for consumption of total dairy, whole milk, low-fat milk, refrigerated yogurt, or pregnant milk powder.

**Conclusion:**

Our results indicated that higher consumption of room-temperature-storage yogurt before pregnancy might be a risk factor for GDM. Further research is warranted to elucidate the underlying associations and mechanisms between dairy consumption and GDM.

## 1 Introduction

Gestational diabetes mellitus (GDM) is defined as any degree of glucose intolerance with onset or first recognition during pregnancy (1). In China, the implementation of “two-child policy” has led to significant rise in proportion of pregnant women with advanced maternal age and the prevalence of GDM showed an upward trend as well (2, 3). A recent meta-analysis involving 79,064 Chinese pregnant women indicated that the pooled prevalence of GDM in mainland China was 14.8% (4). GDM has been linked with a range of adverse perinatal outcomes for both mothers and their offspring, such as caesarean section, preterm delivery, macrosomia, and infant born large for gestational age (LGA) (5, 6). Besides, women with a history of GDM are at higher risk of GDM during subsequent pregnancies, type 2 diabetes mellitus (T2DM), and cardiovascular disease (CVD) in the future (7, 8). Therefore, it is imperative to identify modifiable risk factors for GDM prevention, among which dietary strategies play a vital role (9).

During pregnancy, insulin sensitivity decreases by 50–70%, while insulin secretion increases by 200–250% in order to maintain a euglycemic state (10). As a result, pregnant women are vulnerable to glucose metabolism disorder. Dairy foods, a rich source of nutrients such as protein, lactose, vitamins, calcium and other minerals, could have important impacts on metabolic profile and composition of the gut microbiome (11, 12). It has been suggested to be associated with the prevention of overweight or obesity, CVD, and T2DM (13–15). However, evidence is limited and inconsistent regarding the association between dairy consumption and risk of GDM. In a case–control study conducted in Iran, pre-pregnancy dairy consumption was linked with lower risk of developing GDM (16). Similarly, probiotic yogurt consumption during pregnancy might have protective effects on GDM, and it was also associated with a significant reduction in fasting plasma glucose (17). Incongruous with the above studies, the other two studies found no significant association between dairy consumption or fat-free dairy product consumption and risk of GDM (18, 19). The inconsistent results of these studies might be due to divergences in study design, observed time windows of dairy consumption, type of dairy products, ethnicity of participants and potential confounders.

The existing studies about dairy consumption and risk of GDM mainly focus on the intake during pregnancy, few of which analyzing pre-pregnancy dairy intake. Additionally, most of studies consider total dairy consumption as an independent variable, whether the consumption of a specific type of dairy product is associated with the risk of GDM has not been pinpointed yet. Dairy consumption and the prevalence of GDM vary from country to country, and there were few relevant studies conducted in China yet. Thus, it is essential to investigate their associations among Chinese population.

Using data from a prospective cohort of Chinese women, this study aimed to examine the associations between pre-pregnancy dairy consumption (total dairy, whole milk, low-fat milk, refrigerated yogurt, room-temperature-storage yogurt and pregnant milk powder) and risk of developing GDM in Shanxi, China.

## 2 Materials and methods

### 2.1 Study population

This prospective cohort study was conducted in Taiyuan, Shanxi province between December 2022 and March 2024. Participants were recruited from the Department of Obstetrics in Shanxi Bethune Hospital. Pregnant women who attended their first prenatal visit before 13 weeks’ gestation were invited to participate in this study if they were aged 18–40 years, singleton pregnancy, and had lived in their current residence for at least 1 year. Women with preexisting diabetes, hypertension, mental-health disorders or other major diseases were excluded. All the participants were followed up at each prenatal visit for medical examination until they delivered. The study was approved by the Ethics Committee of Shanxi Bethune Hospital (reference number: YXLL-2023-218). Written informed consent was obtained from all participants at initial enrollment.

**Figure 1:**
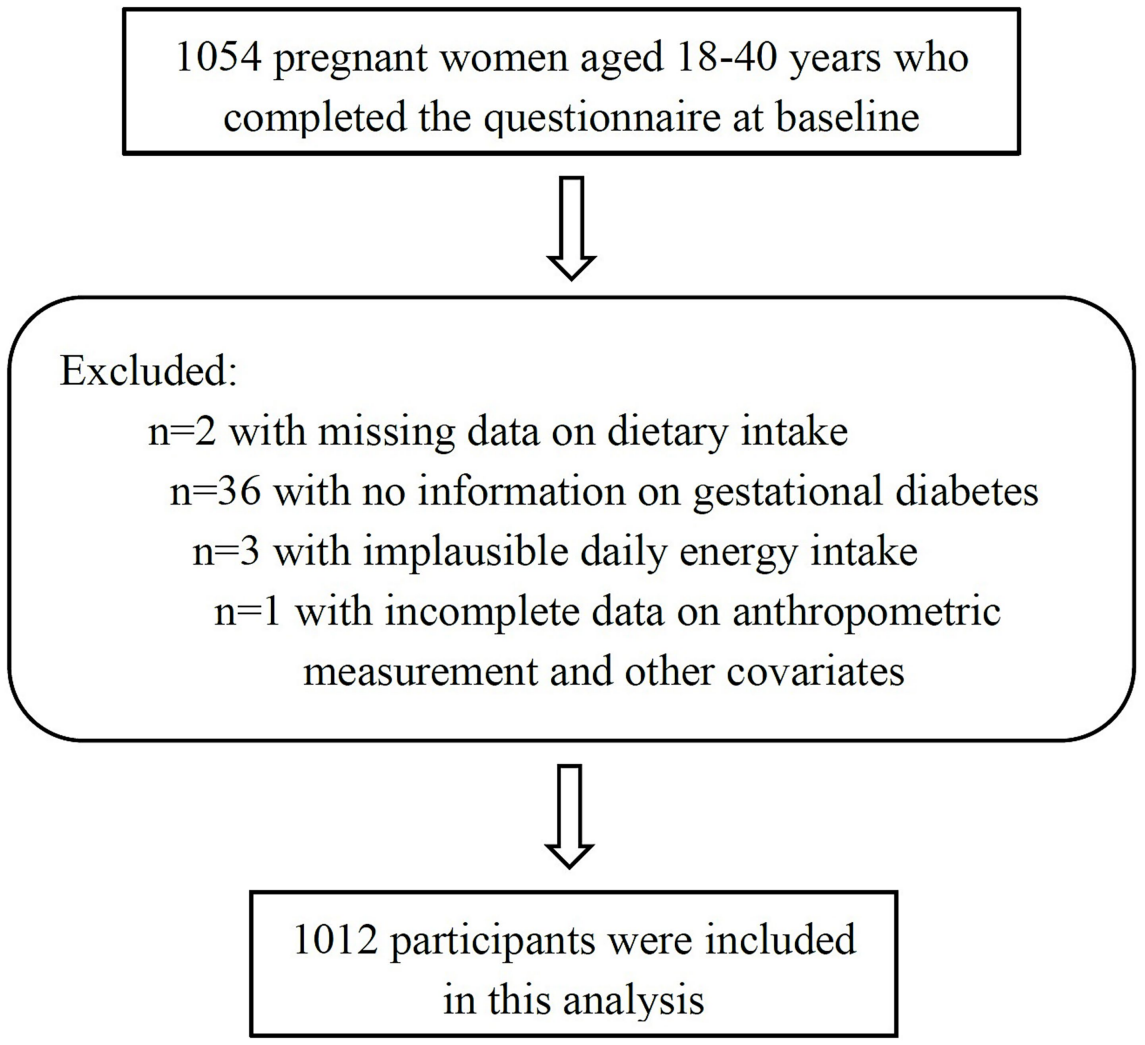
Flow chart of the study sample.

During the study period, 1,054 pregnant women who completed the questionnaire were invited to join the cohort. Participants with missing data on dietary intake (*n* = 2) and gestational diabetes (*n* = 36) were excluded. Three pregnant women with implausible daily energy intake (below 500 or above 4,000 kcal/day) and one with incomplete data on anthropometric measurement and other covariates were further precluded. Finally, the current analysis was based on a sample of 1,012 (Figure 1).

### 2.2 Dietary assessment

The conception date is estimated according to the date of last menstrual period and/or the nuchal translucency scan at the prenatal visit. Maternal dietary information 1 year before pregnancy (1 year before the estimated conception date) and during the first trimester (1–12 weeks of gestation) were obtained at the first prenatal visit (usually 9–12 weeks of gestation) and the following prenatal visit (usually 13–14 weeks of gestation) during the second trimester (13–27 weeks of gestation) respectively, using a modified, semi-quantitative food frequency questionnaire (FFQ), which was shown to be valid among Chinese women (20). The FFQ consisted of 94 food items or groups, covering the most common foods consumed in Northern China, which were assembled into 18 categories (whole grains, refined grains, tubers, vegetables, fruits, nuts, meat, fish and shrimp, animal viscera, eggs, dairy and dairy products, soybeans and its products, fried foods, pickled products, sugar-sweetened beverages, fruit juice, tea and coffee). Participants were required to report the frequency (never, daily, weekly, monthly, yearly) and portion size of the food consumed, with the aid of food models and pictures to increase the accuracy in portion size estimation. During the face-to-face interview, the trained investigators checked FFQs for potentially incorrect responses and clarified with participants when necessary. Dairy and dairy products were divided into whole milk, low-fat milk (including fat-free milk), refrigerated yogurt, room-temperature-storage yogurt (a common type of yogurt that has been heat-treated after fermentation to eliminate live bacteria, allowing it to be safely stored at room temperature for several months without spoiling), and milk powder for pregnant women (a special formula milk powder fortified with vitamins and minerals essential for pregnancy). Daily consumption (g/d) of dairy and dairy products was calculated according to the FFQs.

Dietary consumption data were converted into energy and nutrient intake on the basis of 2004, 2009 and 2018 China Food Composition Table (21–23). Consumption of nutrient supplements was also investigated and computed into nutrients according to the manufacturer instructions.

### 2.3 Evaluation of diet quality

Chinese Healthy Eating Index (CHEI) was used to evaluate pre-pregnancy diet quality of the study participants. Details on the development and calculation of CHEI have been described elsewhere (24). The CHEI was designed as a continuous scoring system, comprising 12 adequacy components (i.e., total grains, whole grains and mixed beans, tubers, total vegetables, dark vegetables, fruits, dairy, soybeans, fish and seafood, poultry, eggs, seeds and nuts) and 5 limitation components (i.e., red meat, cooking oils, sodium, added sugar, and alcohol) with different weightings; the maximum total score is 100. Scoring for the CHEI components is based on the energy density (as amounts per 1,000 calories of intake). Higher CHEI scores indicate better dietary quality. It should be noted that since the original CHEI includes a dairy/dairy products component, we used a modified CHEI score that excluded this component in the multivariable models (resulting in a total score of 95).

### 2.4 OGTT and diagnosis of GDM

At 24–28 weeks of gestation, all of the participants were scheduled for a 75 g oral glucose tolerance test (OGTT) after an overnight fast of 8 to 10 h. Maternal plasma glucose levels were measured in a standardized clinical laboratory via an automatic biochemical analyzer (Backman AU5821, USA). According to the criteria recommended by the International Association of Diabetes and Pregnancy Study Group (IADPSG), GDM was diagnosed if any one of the following conditions was met: fasting plasma glucose ≥ 5.10 mmol/L; 1-h plasma glucose ≥ 10.00 mmol/L; or 2-h plasma glucose ≥ 8.50 mmol/L (25).

### 2.5 Covariates

Basic information on birthdate, ethnicity, place of residence, occupation, education level, per capita monthly household income, parity, age at menarche, history of macrosomia/fetal malformation/fetal death, and family history of diabetes was obtained by structured questionnaire at enrollment. Pre-pregnancy and pregnancy lifestyle information including smoking or passive smoking (yes or no), drinking (g/day), physical activities (minutes/day), screen time (minutes/day), and sleeping duration (hours/day) were also collected.

Pre-pregnancy body weight was self-reported and women’s height was measured at the first prenatal visit. Pre-pregnancy body mass index (BMI) was calculated as pre-pregnancy weight (kg) divided by height squared (m^2^) and was categorized according to standard of Working Group on Obesity in China (WGOC) as underweight (< 18.5 kg/m^2^), normal weight (18.5–23.9 kg/m^2^), overweight (24 kg/m^2^ ≤ BMI < 28 kg/m^2^), or obese (BMI ≥ 28 kg/m^2^) (26). Gestational age was estimated by combining the date of the last menstrual cycle and ultrasound fetal biometric scan provided by an obstetrician.

### 2.6 Statistical analysis

Statistical analyses were performed with SAS procedure (version 9.4, 2018, SAS Institute Inc., Cary, NC, USA) and R software (version 4.4.1, R Development Core Team). A *p*-value < 0.05 was considered statistically significant, except for interaction tests, where *p* < 0.1 was considered significant. Continuous variables were presented as mean ± standard deviation (SD) or median (25th percentile, 75th percentile) according to their normality. Categorical variables were reported as frequency and proportion [n (%)]. Kruskal-Wallis tests were used to compare the difference of nutritional intakes between subgroups of total dairy consumption.

According to the Dietary Guideline for Chinese (DGC-2022), adult women were recommended to consume 300–500 g of dairy and dairy products per day (27). Thus, participants were categorized into three groups according to total dairy consumption (<300 g/day, 300–500 g/day, >500 g/day). The percentages of participants who did not consume any types of whole milk, low-fat milk, refrigerated yogurt, room-temperature-storage yogurt, or pregnant milk powder were approximately 26.0, 77.5, 36.6, 42.7 and 86.7%, respectively. Thus, in our primary analyses we categorized the non-consumers of each subtype of dairy products as a group. For whole milk, refrigerated yogurt and room-temperature-storage yogurt, the daily average consumption of was evaluated and categorized according to tertiles based on the weighted distribution in each consumer group. For low-fat milk, participants were categorized according to the median. In detail, the consumption of whole milk, refrigerated yogurt and room-temperature-storage yogurt were grouped into four categories: 0 g/day, 0–142.9 g/day, 142.9–250.0 g/day, and 250.0–357.1 g/day; 0 g/day, 0–36.0 g/day, 36.0–77.1 g/day and 77.1–180.0 g/day; 0 g/day, 0–35.9 g/day, 35.9–117.1 g/day, and 117.1–205.0 g/day, respectively, whereas the consumption of low-fat milk and pregnant milk powder were grouped into three categories (0 g/day, 0–231.2 g/day and >231.2 g/day) and two categories (0 g/day and >0 g/day), respectively. Then, logistic regression models were conducted to analyze the odds ratio (OR) and 95% confidence intervals (CIs) for the risk of developing GDM compared with the reference group (0 g/day). Each potential confounder was initially considered separately and was kept in the model if the changes of estimates were greater than 10% (28). Three models were performed in this study. In model 1 (crude model), no covariates were included. In model 2, age at enrollment (years), total energy intake (kcal/day), family history of diabetes (yes or no) and per capita monthly household income (Chinese Yuan) were adjusted. As dietary quality and pre-pregnancy weight status might have confounding effects on dairy-GDM relations, CHEI score without dairy (points) and pre-pregnancy BMI (kg/m^2^) were further adjusted in model 3 based on model 2.

Restricted cubic spline (RCS) analyses were conducted to examine the nonlinear associations of dairy/dairy products consumption with odds of GDM. Nonlinearity was tested using the likelihood ratio test. Given the low proportion of those who consumed low-fat milk (22.5%) and pregnant milk powder (13.3%), the RCS analyses were only conducted for total dairy, whole milk, refrigerated yogurt and room-temperature-storage yogurt.

To examine interaction effects, the multiplicative interaction terms were included in the logistic regression models. However, these interaction terms were not significant. Considering the clinical relevance of covariates such as age at enrollment, pre-pregnancy BMI, parity, sleep duration, and passive smoking status, we further performed subgroup analyses to explore whether the effects of dairy consumption on the risk of GDM varied by different subgroups.

## 3 Results

### 3.1 Characteristics of participants

A total of 1,012 pregnant women were included in this study with an average age (at enrollment) of 30.7 ± 3.6 years. Among them, 700 (69.2%) were primiparous and 193 (19.1%) had family history of diabetes. At baseline, 26.5% of the participants were overweight or obese. At 24–28 weeks of gestation, one in eight pregnant women (12.5%) was diagnosed with gestational diabetes mellitus (see Table 1).

**Table 1:** General characteristics of the study participants.

Characteristics	Values [mean ± SD/*n* (%)]
Age at baseline, y	30.7 **±** 3.6
Urban, *n* (%)	963 (95.2)
High education level^a^, *n* (%)	881 (87.1)
High family income^b^, *n* (%)	217 (21.4)
Primiparous, *n* (%)	700 (69.2)
Family history of diabetes, *n* (%)	193 (19.1)
Sleep duration, h/day	7.5 **±** 1.0
Passive smoking, *n* (%)	423 (41.8)
Use of vitamin/mineral supplements, *n* (%)	462 (45.6)
Age at menarche, y	13.5 **±** 1.2
Pre-pregnancy BMI, kg/m^2^	22.3 **±** 3.4
Pre-pregnancy overweight^c^, *n* (%)	268 (26.5)
OGTT-fasting, mmol/L	4.4 **±** 0.4
OGTT-1-h, mmol/L	7.4 **±** 1.6
OGTT-2-h, mmol/L	6.5 **±** 1.4
GDM, *n* (%)	126 (12.5)

BMI, body mass Index; OGTT, oral glucose tolerance test; GDM, gestational diabetes mellitus; SD, standard deviation.

a Bachelor degree or above.

b Per capita monthly household income ≥ 8,000 CNY (Chinese Yuan).

c Pre-pregnancy BMI ≥ 24.0 kg/m^2^ (26).

### 3.2 Pre-pregnancy nutritional intakes by consumption of total dairy

As shown in Table 2, compared with participants who consumed total dairy below recommendation (300 g/day), those who consumed within (300–500 g/day) and above (>500 g/day) recommendation tended to consume more whole grains (*p* = 0.01), total vegetables (*p* = 0.03), eggs (*p* < 0.001), red meat (*p* = 0.01), and seeds and nuts (*p* < 0.001). Among all the participants, those who consumed 300–500 g/day of total dairy had the highest CHEI score of 62.6 points. In addition, participants who consumed total dairy within and above recommendation had higher energy intake than those who consumed below recommendation, as well as other nutrients including protein, fat, fiber, calcium, iron, and folate (all *p* < 0.05). By contrast, women who consumed total dairy below recommendation had highest carbohydrate intake than the other groups (*p* < 0.001).

**Table 2:** Nutritional intakes of participants at the pre-pregnancy according to the consumption of total dairy.^a^

Consumption	Total dairy intake (g/day)			*p*
	<300 (*n* = 551)	300–500 (*n* = 404)	>500 (*n* = 57)	
Food groups				
Total grains, g/day	247.4 (201.0, 296.2)	259.7 (209.0, 319.3)	252.7 (219.0, 325.2)	0.09
Whole grains, g/day	63.5 (35.9, 95.3)	69.1 (41.0, 107.0)	69.3 (46.0, 115.5)	0.01
Tubers, g/day	79.3 (50.0, 126.3)	92.9 (53.2, 135.7)	88.1 (56.0, 156.3)	0.15
Total vegetables, g/day	169 (119.9, 234.6)	186.5 (126.1, 256.5)	205.4 (148.0, 256.3)	0.03
Dark vegetables, g/day	98.0 (67.0, 131.1)	104.1 (70.4, 131.6)	105.7 (75.1, 141.4)	0.41
Fruits, g/day	200.0 (200.0, 230.0)	200.0 (200.0, 230.0)	230.0 (200.0, 230.0)	0.06
Soybeans, g/day	12.1 (6.0, 19.5)	13.0 (7.3, 19.3)	15.0 (8.9, 21.4)	0.23
Fish and seafood, g/day	40.0 (14.0, 104.0)	47.4 (19.0, 99.5)	63.9 (28.3, 145.7)	0.07
Poultry, g/day	13.2 (0.0, 30.0)	16.1 (1.9, 30.0)	22.6 (6.5, 41.3)	0.13
Eggs, g/day	60.0 (51.4, 62.7)	60.9 (60.0, 62.8)	62.7 (60.0, 68.3)	<0.001
Red meat, g/day	40.0 (27.0, 59.7)	46 (31.3, 65.7)	47.0 (28.6, 71.8)	0.01
Seeds and nuts, g/day	2.3 (0.0, 10.5)	6.5 (0.7, 12.9)	10.0 (0.7, 17.1)	<0.001
SSB, ml/day	83.8 (0.0, 180)	88.7 (0.0, 175)	64.2 (0.0, 251.8)	0.87
CHEI score	59.8 (53.9, 63.9)	62.6 (57.8, 66.1)	61.7 (57.1, 64.7)	<0.001
Nutrients				
Energy intake, kcal	1737.7 (1458.5, 2044.7)	1982.0 (1699.5, 2,295)	2222.5 (1951.1, 2653.6)	<0.001
Protein, % of energy	16.7 (15.1, 19.0)	16.8 (15.7, 18.7)	17.7 (16.1, 19.9)	0.049
Fat, % of energy	20.5 (18.0, 23.3)	22.7 (20.8, 24.7)	23.0 (21.1, 25.2)	<0.001
Carbohydrate, % of energy	62.5 (58.7, 66.6)	60.8 (57.0, 63.5)	60.0 (55.5, 62.7)	<0.001
Fiber, g/day	15.1 (11.7, 18.9)	17.0 (13.0, 21.0)	18.2 (14.6, 23.9)	<0.001
Calcium, mg/day	630.9 (495.3, 793.0)	864.1 (755.4, 1022.3)	1183.2 (1066.6, 1438.7)	<0.001
Iron, mg/day	23.4 (17.0, 32.1)	28.0 (20.0, 34.4)	33.2 (24.7, 46.3)	<0.001
Folate, μg/day	353.0 (256.7, 653.8)	546.2 (305.8, 705.3)	653.8 (301.5, 782.2)	<0.001
SFA, g/day	10.0 (7.6, 12.3)	14.1 (12.4, 16.6)	18.1 (16.4, 20.8)	<0.001
MUFA, g/day	8.5 (6.6, 11.3)	11.3 (9.4, 14)	13.2 (11.6, 16.7)	<0.001
PUFA, g/day	4.3 (3.0, 6.3)	5.6 (3.9, 7.3)	6.6 (5.1, 9.8)	<0.001

SSB, sugar-sweetened beverages; CHEI, Chinese Healthy Eating Index; SFA, saturated fatty acid; MUFA, monounsaturated fatty acid; PUFA, polyunsaturated fatty acid.

a Values were presented as median (25th percentile, 75th percentile), and Kruskal-Wallis test was used to compare the difference of nutritional intakes between subgroups of total dairy consumption.

### 3.3 The association between pre-pregnancy dairy consumption and GDM

Logistic regression models for associations of total dairy and different type of dairy products consumption with odds of GDM are presented in Table 3. Compared with non-consumers of room-temperature-storage yogurt, women in the highest tertiles of consumption had 2.11-fold higher odds of GDM after adjusting for age at enrollment, energy intake, family history of diabetes, per capita monthly household income in model 2 (OR: 2.11, 95% CI: 1.02–4.13), and this association remained significant when CHEI score and pre-pregnancy BMI were further included in model 3 (OR: 2.64, 95% CI: 1.25–5.38). There was a positive trend (not significant) between the consumption of room-temperature-storage yogurt and risk of GDM in the final model (*P* for trend = 0.059). No statistically significant linear association/trend between consumption of total dairy, whole milk, low-fat milk, refrigerated yogurt, or pregnant milk powder and odds of GDM was observed after adjustment for potential covariates. In the RCS analyses, no nonlinear association between dairy consumption and odds of GDM was found (Supplementary Figure 1).

**Table 3:** Association between consumption of dairy/dairy products and gestational diabetes mellitus by logistic models.^a^

Dairy products	Model 1^b^	Model 2^c^	Model 3^d^
	OR (95%CI)	OR (95%CI)	OR (95%CI)
Total dairy (g/day)			
<300	1.00	1.00	1.00
300—500	1.17 (0.76, 1.77)	1.09 (0.70, 1.70)	1.15 (0.73, 1.81)
>500	0.86 (0.29, 2.09)	0.71 (0.23, 1.84)	0.86 (0.27, 2.26)
*P* for trend	0.70	0.69	0.76
Whole milk (g/day)			
0	1.00	1.00	1.00
0—142.9	0.97 (0.53, 1.75)	0.98 (0.53, 1.80)	1.004 (0.53, 1.89)
142.9—250.0	1.14 (0.69, 1.92)	1.07 (0.63, 1.83)	1.12 (0.64, 1.99)
250.0—357.1	1.28 (0.49, 3.01)	1.06 (0.39, 2.56)	1.04 (0.38, 2.60)
*P* for trend	0.88	0.99	0.97
Low-fat milk (g/day)			
0	1.00	1.00	1.00
0—232.1	1.69 (0.48, 4.66)	0.98 (0.26, 2.95)	0.91 (0.23, 2.84)
>232.1	1.19 (0.28, 3.61)	1.12 (0.25, 3.60)	0.78 (0.16, 2.76)
*P* for trend	0.63	0.98	0.93
Refrigerated yogurt (g/day)			
0	1.00	1.00	1.00
0—36.0	0.90 (0.51, 1.54)	1.03 (0.58, 1.81)	0.97 (0.54, 1.74)
36.0—77.1	0.75 (0.41, 1.33)	0.78 (0.42, 1.41)	0.82 (0.43, 1.52)
77.1—180.0	0.82 (0.47, 1.40)	0.81 (0.45, 1.42)	0.86 (0.48, 1.52)
*P* for trend	0.78	0.77	0.92
Room-temperature-storage yogurt (g/day)			
0	1.00	1.00	1.00
0—35.9	0.98 (0.44, 1.96)	1.22 (0.54, 2.48)	1.44 (0.63, 3.00)
35.9—117.1	1.12 (0.56, 2.08)	1.13 (0.55, 2.16)	1.21 (0.57, 2.38)
117.1—205.0	1.88 (0.94, 3.53)	2.11 (1.02, 4.13)	2.64 (1.25, 5.28)
*P* for trend	0.30	0.21	0.059
Pregnant milk powder (g/day)			
0	1.00	1.00	1.00
>0	1.18 (0.34, 3.12)	1.41 (0.40, 3.90)	1.48 (0.41, 4.24)
*P* for trend	0.77	0.54	0.50

a Values are presented as odds ratio (OR) and 95% confidence interval (95%CI).

b Model 1: crude model.

c Model 2: adjusted for age at enrollment, total energy intake, family history of diabetes, per capita monthly household income.

d Model 3: adjusted for age at enrollment, total energy intake, family history of diabetes, per capita monthly household income, CHEI score, and pre-pregnancy BMI.

### 3.4 Subgroup analyses

**Figure 2:**
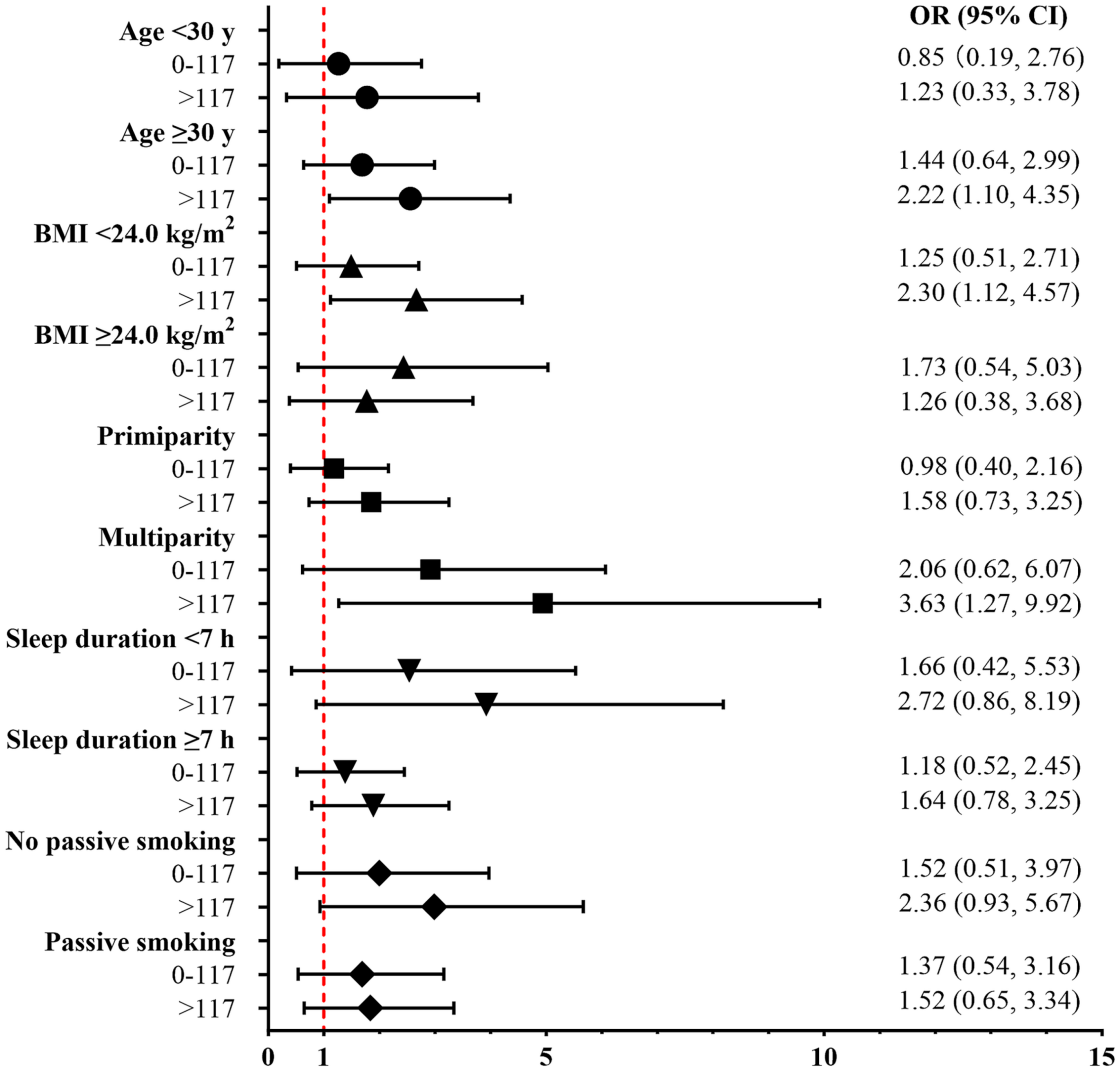
Association between consumption of room-temperature-storage yogurt and gestational diabetes mellitus in subgroup analyses. Consumption of room-temperature-storage yogurt was categorized into three groups: 0 g/day, >0 and ≤117 g/day, and >117 g/day according to the median of consumption. Logistic regression analyses compared OR with the reference group (0 g/day). Age at enrollment, total energy intake, family history of diabetes, per capita monthly household income, CHEI score, and pre-pregnancy BMI were adjusted in the logistic models except for the subgroup factors.

Subgroup analyses revealed that the association between room-temperature-storage yogurt consumption and risk of GDM might be affected by maternal age, pre-pregnancy BMI, and parity. For women who were 30 years and above, whose pre-pregnancy BMI was under 24.0 kg/m^2^ and who were multiparas, consuming more room-temperature-storage yogurt (>117 g/day) were associated with 2.22-fold (OR: 2.22, 95% CI: 1.10–4.35), 2.30-fold (OR: 2.30, 95% CI: 1.12–4.57) and 3.63-fold (OR: 3.63, 95% CI: 1.27–9.92) higher odds of developing GDM (see in Figure 2). In other subgroups, there was no significant association between room-temperature-storage yogurt consumption and risk of GDM.

## 4 Discussion

The present study demonstrated that consumption of room-temperature-storage yogurt 1 year before pregnancy was positively associated with the risk of GDM among Chinese women. This association was more obvious among women over the age of 30, with BMI under 24.0 kg/m^2^ and who were multiparas. Nevertheless, pre-pregnancy consumption of total dairy, whole milk, low-fat milk, refrigerated yogurt and pregnant milk powder showed no significant associations with GDM.

In China, a variety of dairy products, equivalent to 300–500 mL of liquid milk per day, are recommended in the DGC-2022 (27). However, the average total dairy consumption was 273.7 g/day in our study, below the lower limit of recommendation. Although dairy products have been more available and intakes of dairy have been increasing in the past decades, dairy consumption in China remains low compared to other global regions including Europe and the U.S (29). This might be attributed to several factors such as rising prices of dairy products, high proportion of lactose intolerance in Asian populations, concerns of food safety and a traditional plant-based dietary pattern (29, 30). In addition, we found that participants whose total dairy consumption reached the recommendation had higher dietary quality than those below the recommendation. Accordingly, another study observed that pregnant women who have a high adherence to the MedDiet also have higher consumption of dairy products (18). These results indicated that women consumed more dairy products might have higher nutritional literacy and pay more attention to their diet quality.

To our knowledge, this is the first prospective cohort study investigating the association of pre-pregnancy dairy consumption with GDM among Chinese women. The lack of associations between total dairy consumption and risk of GDM was consistent with a prior study reporting null findings in the United States (19). However, another case–control study by Pouladi et al. (16) indicated protective effects of pre-pregnancy dairy consumption on GDM. Evidence suggested that dairy products containing specific components, such as calcium, vitamin D, magnesium, potassium, and whey protein, have a beneficial impact on glucose metabolism (31–33). These specific components might increase insulin secretion/sensitivity and reduce insulin resistance (34, 35). In our study, the relative lower total dairy consumption (273.7 g/day) and incidence of GDM (12.5%) might make it more difficult to discover significant association between total dairy and risk of GDM. Furthermore, if there are variations in the associations within dairy types, the mixture of all dairy types with different nutrient compositions might lead to the null findings as well.

In this study, neither consumption of whole milk nor low-fat milk was associated with risk of GDM. Similar results were observed in a previous study among Spanish women, where the fat-free milk consumption during pregnancy was not associated with risk of GDM (18). A recent meta-analysis concluded that low-fat milk consumption was associated with 4% lower odds of T2DM whereas whole milk consumption showed no association with T2DM risk (13). However, there was evidence indicated that consumption of full-fat versus low-fat dairy was associated with a lower incidence of diabetes (36). Whether milk fat could be detrimental, due to its high proportion of saturated fatty acids (SFAs), remains controversial. Whole fat dairy foods contain some bioactive fatty acids and have been demonstrated to have favorable effects on cardiometabolic health (37, 38), which might counteract part of the harmful impacts of SFAs. Since GDM and T2DM share pathophysiological mechanisms, low-fat/ whole milk consumption may also have homologous effects on development of GDM. Prospective cohort studies with large sample or mechanism studies are still warranted.

Several previous studies have showed a protective effect of yogurt consumption on glucose metabolism of pregnant women or GDM risk (17, 39, 40). These studies mainly focused on probiotic yogurt, which can modify the gut microbiome composition and have been shown to achieve better control of blood glucose in GDM patients (41). Furthermore, the nutrients in yogurt may be more bioavailable through fermentation process than in other dairy products (42). In our study, yogurt was further subdivided into refrigerated yogurt and room-temperature-storage yogurt. Inconsistent with previous study, we found that consumption of room-temperature-storage yogurt was associated with higher odds of GDM. Several reasons might explain this positive association. Room-temperature yogurt is under a more stringent sterilization process and can be stored at room-temperature for several months, causing a significant decrease in quantity and viability of probiotic bacteria (43). Besides, most room-temperature yogurts in China, e.g., *Ambrosial* and *Chunzhen*, are sweetened with sugar or other sweeteners to improve its palatability nowadays, which might elevate blood glucose of pregnant women (44). Lastly, a slight Maillard reaction might occur in the sterilization and subsequent storage process of room-temperature-storage yogurt. The Maillard reaction could produce substances known as advanced glycation end products (AGEs), which might be associated with inflammatory responses and oxidative stress, further resulting in insulin resistance and diabetes (45, 46). In further subgroup analysis, the positive association between room-temperature-storage yogurt consumption and risk of GDM was only observed in women who were 30 years and above, whose pre-pregnancy BMI was lower than 24.0 kg/m^2^ and who were multiparas. It is well established that advanced maternal age and high pre-pregnancy BMI are risk factors of developing GDM (47). Multiparas are usually older than primiparas, thus, the incidence of GDM is high in multiparas as well. However, the sample size of subgroup with pre-pregnancy BMI ≥ 24.0 kg/m^2^ was too small owing to the relative lower prevalence of overweight or obesity in our study, so we found no significant associations in this subgroup.

Several limitations of the current study should be acknowledged. Samples were all recruited from hospitals rather than the community, which may affect the generalizability of this study. Secondly, the use of memory-based FFQ for dietary assessment is subject to inherent limitations. Although a food diary may provide more accurate dietary data, its implementation is resource-intensive and challenging in a large-scale epidemiological study. Therefore, the FFQ was considered the most feasible instrument for the current sample size. Low proportion of participants who consumed low-fat milk and pregnant milk powder in this study made it difficult to find significant associations, and we should enlarge our study sample in further analysis. Furthermore, associations between consumption of one specific dairy product and risk of GDM were analyzed without consideration of the consumption of other types of dairy products. For example, this means that it could be possible for some women who consumed both low-fat and whole milk, and they might have mutual impacts on risk of GDM. Finally, several confounders, such as gestational weight gain, social capital, depressive symptoms, were not considered in this study. Existing evidence has indicated that high gestational weight gain and depression symptoms might be associated with increased risk of GDM (48, 49). Whether these confounders could influence the associations between dairy consumption and GDM deserves further exploration.

Despite the limitations, our study has several strengths. We assessed the dairy consumption 1 year before pregnancy rather than during pregnancy, which accurately clarified the sequence of exposure and outcome, and the prospective cohort study design enhanced the strength of evidence. Our study systematically analyzed the impacts of total dairy consumption as well as different types of dairy products on the risk of GDM, enriching the results of previous studies. Additionally, many potential confounders, including maternal age at enrollment, family history of diabetes, total energy intake, pre-pregnancy BMI and dietary quality, were considered for adjustment simultaneously and subgroup analyses were conducted. Overall, on the basis of robust models we constructed, this study might provide women preparing for pregnancy with guidance on reasonable selection of dairy products for GDM prevention.

## 5 Conclusion

Our study suggests that consumption of room-temperature-storage yogurt was associated with a higher risk of developing GDM among Chinese women, and maternal age, pre-pregnancy BMI and parity might influence this association. Nonetheless, consumption of total dairy and other types of dairy products was not associated with GDM risk. More studies are warranted to elucidate the role of dairy consumption or specific dairy foods in the development of GDM among Chinese population.

## Data Availability

The raw data supporting the conclusions of this article will be made available by the authors, without undue reservation.
