# Supplementary material for: Effects of pre-pregnancy dairy consumption on gestational diabetes mellitus: a prospective cohort study among Chinese women
Source: Front Nutr. 2026 Apr 29;13:1769975. doi: 10.3389/fnut.2026.1769975 (PMC13167434; doi:10.3389/fnut.2026.1769975)
Supplement: Supplementary file 1 [file Image_1.pdf]

Supplementary Material

1 Supplementary Figures

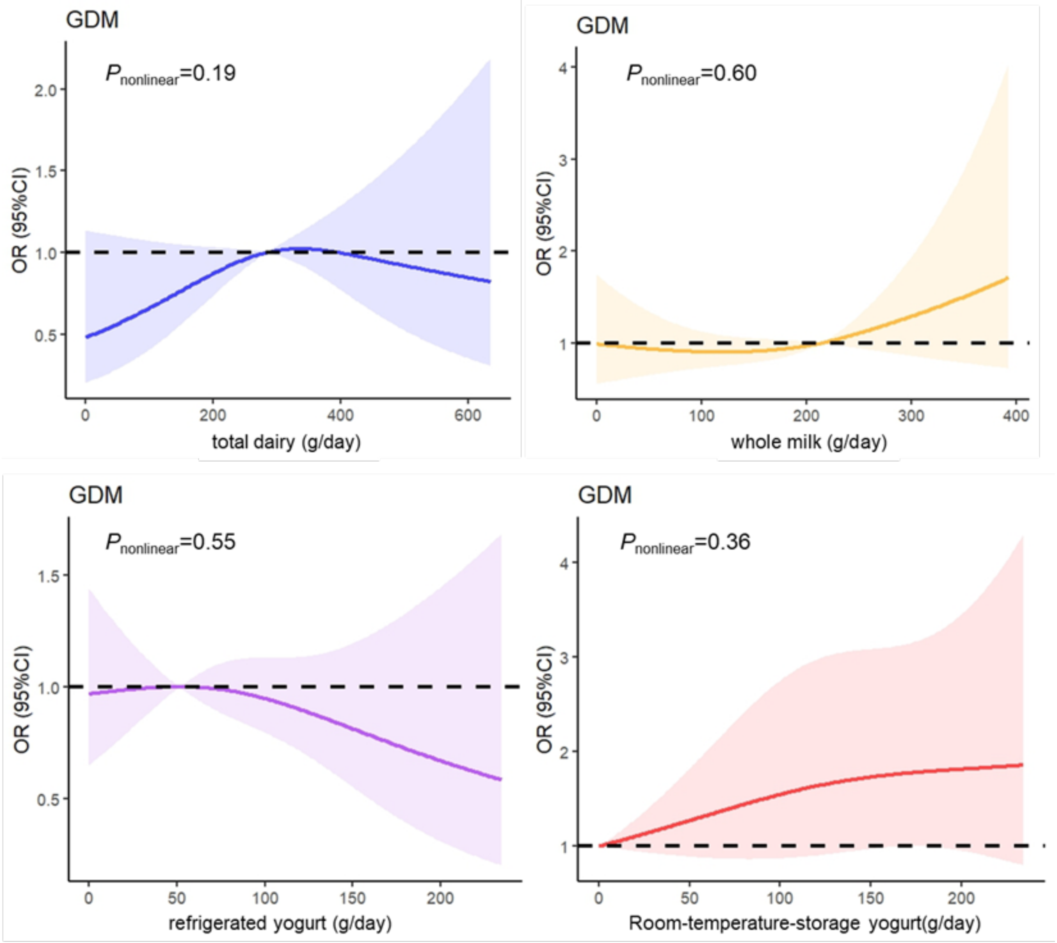

Supplementary Figure 1 Nonlinear association between consumption of dairy/dairy products and gestational diabetes mellitus (GDM) by restricted cubic spline (RCS) models [a].

[a] RCS models were adjusted for age at enrollment, total energy intake, family history of diabetes, per capita monthly household income, CHEI score, and pre-pregnancy BMI.
